# Association Between Diet Quality and Risk of Ovarian and Endometrial Cancers: A Systematic Review of Epidemiological Studies

**DOI:** 10.3389/fonc.2021.659183

**Published:** 2021-05-18

**Authors:** Yu-Hua Zhang, Zhuo Li, Ming-Zi Tan

**Affiliations:** Department of Gynecology, Cancer Hospital of China Medical University, Liaoning Cancer Hospital & Institute, Shenyang, China

**Keywords:** diet quality, ovarian cancer, edometrial cancer, risk factors, epidemiological studies

## Abstract

**Objectives:**

The relationship between diet quality indices and risk of ovarian and endometrial cancers were unclear. We aimed at conducting a systematic review to evaluate the epidemiological evidence.

**Methods:**

Embase, PubMed, Web of Science and Scopus databases were searched for eligible studies up to December 2020. Epidemiological studies reported the association of the diet quality with risk of ovarian and endometrial cancers were evaluated.

**Results:**

Eleven eligible studies were identified, of which six studies were case-control studies, four were cohort studies, and one was case-cohort study. All studies were considered as high-quality with low risk of bias. Seven studies evaluated the association of diet quality with risk of ovarian cancer. Four studies reported null association for diet quality indices such as Healthy Eating Index (HEI)-2005, HEI-2010, Mediterranean Diet Score (MDS) and Recommended Foods Score (RFS). Two studies reported significantly inverse association for Alternate Healthy Eating Index (AHEI)-2010 and Healthy Diet Score (HDS) indices. One study reported significantly increased risk of ovarian cancer associated with higher level of Dietary Guidelines for Americans Index. Dose-response analysis showed pooled relative risks of 0.98 (95%Cl: 0.95, 1.01) and 0.94 (95%Cl: 0.77, 1.13) for each 10 points increase in the HEI-2005 and AHEI-2010 indices. Seven studies evaluated the association of diet quality with risk of endometrial cancer. Three studies reported significantly inverse association of diet quality as assessed by the MDS and Diet Score Quintiles with risk of endometrial cancer. Four studies reported null association for other diet quality indices including HEI-2005, HEI-2010, RFS and HDS. Dose-response analysis showed a pooled relative risk of 0.87 (95%CI: 0.81, 0.93) for one unit increment of the MDS.

**Conclusion:**

This study suggests little evidence on the association between diet quality and risk of ovarian cancer. Adherence to high quality diet, as assessed by MDS, might be associated with lower the risk of endometrial cancer.

## Introduction

Ovarian and endometrial cancers are two most common gynecological cancers and the leading cause of death due to gynecological cancer among women in Western countries (1). According to the GLOBOCAN cancer statistics, the estimated numbers of new cases due to ovarian and endometrial cancers worldwide were 295,414 and 382,069 in 2018, respectively (1). Thus, the identification of modifiable risk factors has continued being of clinical and public health importance to prevent ovarian and endometrial cancers.

Risk factors for ovarian cancer include absence of pregnancy, early age of menarche, late age at menopause, family history of ovarian cancer, smoking and benign gynecological conditions (2). Diet may play an important role in the etiology of ovarian cancer, but the associations between ovarian cancer risk and dietary factors such as black tea, milk, lactose and calcium were supported by weak evidence (2, 3). Well-established risk factors of endometrial cancer include nulliparous, higher body mass index and waist-to-hip ratio (4). However, dietary factors for ovarian cancer are still not apparent. Increased intake of coffee, monounsaturated fatty acid and fiber may be associated with lower risk of endometrial cancer, while western style pattern dietary intake may be associated with higher risk of endometrial cancer (4).

Although it is important to understand the role of individual dietary constituents, foods are not consumed in isolation. Diet quality indices such as the Healthy Eating Index-2005 (HEI-2005), Healthy Eating Index-2010 (HEI-2010), Alternate Healthy Eating Index-2010 (AHEI-2010) and Mediterranean diet score (MDS)/Alternate Mediterranean Diet Score (aMDS) address the complexity of the diet and the likely interaction between multiple diet components (5). Despite the various differences between these diet indices, a commonality is that they assess intake of desirable food groups such as fruits, vegetables, whole grains, nuts, and legumes (5). Diets that score highly on the HEI and AHEI were associated with a significant reduction in the risk of all-cause mortality, cardiovascular disease, cancer, type 2 diabetes, and neurodegenerative disease (6). The highest-quality diet as assessed by MDS was associated with reduced risks of breast and upper gastrointestinal cancers (7, 8). Previous epidemiological studies have evaluated the association between a variety of diet quality indices such as HEI, AHEI, MDS and other diet scores and risk of ovarian and endometrial cancers with inconsistent results (9–19). Several studies indicated that high quality diet was significantly associated with lower risk of these cancers (10, 12–14), whereas other studies suggested the null association (9, 11, 15–18). A US cohort study even reported an increased risk of ovarian cancer associated with higher adherence of the fifth edition of the Dietary Guidelines for Americans (19).

A thorough understanding of the relation between diet quality and risk of ovarian and endometrial cancers could facilitate the development of more effective strategy and recommendation on cancer prevention in women. Thus, we aimed at conducting a systematic review to evaluate the association between diet quality and risk of ovarian and endometrial cancers.

## Materials and methods

This study was performed in adherence to PRISMA guidance (**Supplementary Document 1**) (20). Two researchers (YHZ and ZL) independently conducted the information collection including the literature search, study selection and information extraction. Whenever there is a discrepancy between the two authors, they first try to resolve it by an internal discussion; if consensus still cannot be reached, a third reviewer (MZT) acted as an arbitrator to make the final decision.

### Literature Search

The Embase, PubMed, Web of Science and Scopus databases were searched up to Dec 2020, using the Mesh terms as well as the following words: (“diet quality” OR “diet score” OR “diet index” OR “healthy eating index” OR “Mediterranean diet” OR “dietary guidelines” OR “lifestyle index”) AND (ovarian OR ovary OR endometrial OR endometrium) AND (cancer OR tumor OR neoplasm OR carcinoma). We also searched Google Scholar for grey literature. Language of publication was restricted to English. Besides, we screened the reference lists of relevant studies to identify other potential studies. The search strategies were presented in **Supplementary Document 2**.

### Study Selection

The inclusion criteria were as follows: (1) Study design should include cohort study, case-cohort or case-control study; (2) Studies should report diet quality indices as exposure of interest, such as HEI-2005, HEI-2010, AHEI-2010, MDS, other diet quality indices; (3) Studies should report ovarian or endometrial cancer as the outcome of interest; (5) Studies should report relative risk (RR), risk ratio, rate ratio, hazard ratio, odds ratio with the corresponding 95% confidence interval (CI) as the measure of association. When two or more studies covered the same study population, we selected the most recent one, or the most informative study, or extracted information from both studies as appropriate.

### Data Extraction

A structured data extraction form was used to collect the study-specific information, which included the first author of the study, year of publication, study design, study location, study population, size of cohort/number of controls, age at baseline, exposures, exposure assessment methods, outcomes, outcome ascertainment, number of cases, follow-up time, risk estimates, and confounder adjustment. When there were multiple risk estimates in a study, we extracted the one most appropriately adjusted for the confounders in the analysis.

### Study Quality Assessment

The study quality was assessed by two researchers (YHZ and ZL) independently. The Newcastle-Ottawa Scale, which was developed to evaluate the quality of observational studies, was used as the assessment tool (21). A study was judged on three broad perspectives: the selection of the study groups; the comparability of the groups; and the ascertainment of either the exposure or outcome of interest for case-control or cohort studies respectively. “High” quality choices were assigned with a maximum of two stars for items in “comparability” category, and with one star for other numbered items such as “selection of groups” and “ascertainment of the outcome or exposure”. Based on the criteria in previous studies (22, 23), a study with NOS score of ≥7 was considered as the high-quality study.

### Statistical Analysis

Due to various types of dietary quality indices, as well as different ranges and categories of these indices in the population, there was significant heterogeneity across the studies regarding the risk estimates for highest category compared with the lowest category of dietary quality indices. Thus, we only attempted to conduct dose-response meta-analysis to pool the study-specific risk estimates for same dietary indices when more than one study reported the dose-response results. A dose-response meta-analysis may help reduce the heterogeneity, address the inconsistency of the individual studies and add further quantitative information for the review. Compared with risk estimate from a single study, a dose-response meta-analysis may tend to provide relatively precise risk estimates when the heterogeneity was low. The dose-response relation was estimated by using generalized least squares trend estimation, which was proposed by Greenland and Longnecker (24). We assigned the median value in each category of dietary indices to the corresponding RR for each study (25). If medians were not reported, we estimated approximate medians by using the midpoint of the lower and upper bounds (25). If the highest or lowest category of the studies was open-ended, we considered the range of the highest or lowest category to be equivalent to the difference in the closest adjacent category (25). We evaluated the heterogeneity across studies by using the I^2^ statistic (26). Statistical heterogeneity was indicated at an I^2^ of more than 50% (26). Statistical analyses were conducted using Stata 14.0.

## Results

### Study Selection

**Figure 1:**
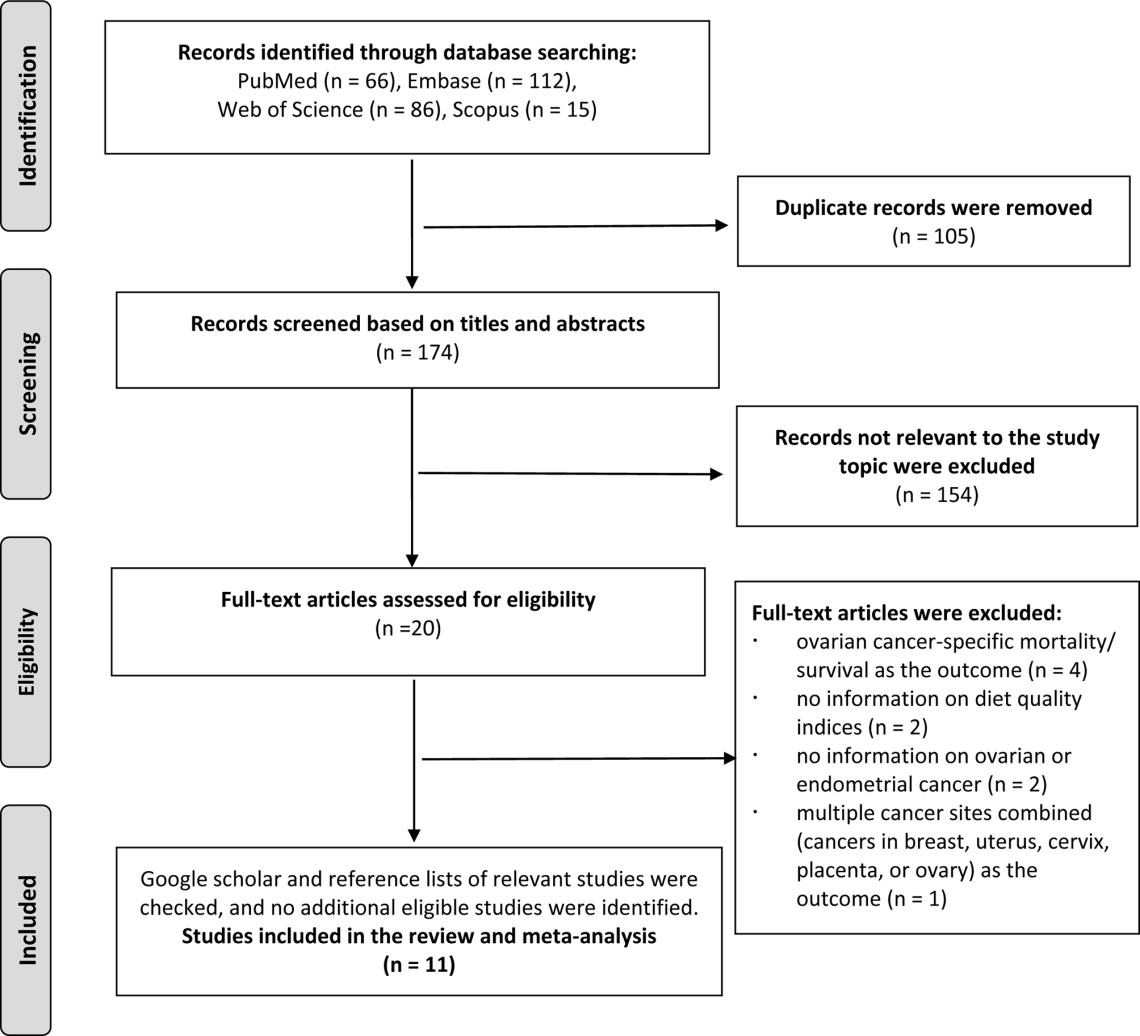
Flow diagram of study selection.

The literature search identified 279 total records from Embase, PubMed, Web of Science and Scopus databases. After we excluded 105 duplicates, 174 records remained for further screening. After screening the titles and abstracts, 154 non-relevant records were excluded, and 20 records were identified for further full-text review. During the full texts review stage, four studies were excluded because of reporting ovarian cancer-specific mortality/survival in women with ovarian cancer (27–30); two studies were excluded because of not reporting information on diet quality indices (31, 32); two studies were excluded because of not reporting information on ovarian or endometrial cancer (33, 34); one study was excluded because of reporting multiple cancer sites combined (cancers in breast, uterus, cervix, placenta, or ovary) as the outcome (35); Finally, we included eleven studies in the review (9–19) (**Figure 1**).

### Study Characteristics

As shown in **Table 1**, among the eleven included studies, six studies were case-control studies, four were cohort studies, and one was case-cohort study. Most of the studies were conducted in Western countries, including US (n=8), Italy (n=2) and Canada (n=1). All the studies collected the diet information by food-frequency questionnaires. The diet quality was assessed by HEI-2005, HEI-2010, AHEI-2010, MDS/aMDS, DASH, Recommended Foods Score (RFS), Healthy Diet Score (HDS) and other diet quality indices. This study included 2626 and 3998 cases of ovarian and endometrial cancers, respectively. Information on cancer diagnosis was obtained from a variety of data sources including medical record review, patient contact, cancer or death registries. Most of the included studies reported risk estimates adjusted for the most potential confounders or risk factors such as age, body mass index, total energy intake, family history and smoking, etc. According to assessments from the Newcastle-Ottawa Scale, all the studies were considered as relatively high quality with low risk of bias (**Supplementary Document 3**).

**Table 1:** Characteristics of the studies included in the review.

Study	Country	Design	Study population	Age	Diet quality index^1^	Dietary method	Follow-up & outcome assessment	Adjustments
Arthur et al. (9)	US	Cohort study	108,136 postmenopausal women	50 to 79 years	Diet score of 6 dietary components (cereal fiber, red and processed meat, the ratio of polyunsaturated to saturated fat, trans-fats, glycemic load, and fruits and vegetables	122-item self-administered food frequency questionnaire	Median follow-up of 17.9 years; 904 ovarian cancer cases and 1,435 endometrial cancer cases; Cancer diagnoses and tumor characteristics were adjudicated centrally by trained physicians, who reviewed medical records and pathology reports	Age at entry, education, nonalcohol energy intake, ethnicity, age at menarche, parity, combined estrogen and progesterone therapy, unopposed estrogen therapy, oral contraceptive use, family history of endometrial or ovarian cancer, age at menopause, physical activity, alcohol consumption, body mass index, and smoking
Arthur et al. (12)	Canada	Case-cohort design	2735 women	Median age for cohort was 58 years	Healthy Diet Score including information on intake of cereal fiber, red and processed meat, margarine, and fruits and vegetables.	166-item self-administered food frequency questionnaire	Median follow-up of 11 years; 100 ovarian cancer cases and 177 endometrial cancer cases	Stratified by age at entry and adjusted for education, non-alcohol energy intake, smoking status, alcohol intake, body mass index, diet score, physical activity, age at menarche, parity, menopause, hormone replacement therapy use, oral contraceptive use
Qin et al. (10)	US	Population-based case-control study	415 ovarian cases and 629 controls; Cases were newly diagnosed and histologically confirmed invasive epithelial ovarian cancer from cancer registries or hospitals’ gynecologic oncology departments; Controls had no previous history of ovarian cancer, and had not had previous bilateral oophorectomy, identified by list-assisted, random-digit dialing	20 to 79 years	Healthy Eating Index -2005, Healthy Eating Index -2010, and Alternative Healthy Eating Index -2010	Self-administered Block 2005 food frequency questionnaire for 110 foods and beverages	NA	Age, region, education, parity, oral contraceptive use, menopause status, tubal ligation status, first-degree family history of breast/ovarian cancer, body mass index, physical activity, and total energy intake.
Ricceri et al. (13)	Italy	a case-control study	297 women with newly diagnosed endometrial cancer and 307 controls	40 to 74 years	Mediterranean Diet Score	Validated food frequency questionnaire	NA	Age, age at menarche, parity, oral contraceptive use, menopausal status, use of hormone replacement therapy, body mass index, physical activity, education, smoking status, and total energy intake.
Filomeno et al. (14)	Italy	Pooled analysis of three hospital based case-control studies	1411 endometrial cancer cases and 3668 controls	Median age of cases was 61 year; median age of controls was 57 years	Mediterranean Diet Score	Validated food frequency questionnaire	NA	Age, study center, year of interview, education, tobacco smoking, body mass index, age at menopause, age at menarche, parity, oral contraceptive use, hormone-replacement therapy use, history of hypertension, diabetes and total energy intake.
George et al. (15)	US	Cohort study	84,415 postmenopausal women	50 to 79 years	Healthy Eating Index -2010, Alternative Healthy Eating Index -2010, alternate Mediterranean Diet Score, and Dietary Approaches to Stop Hypertension	Self-administered, validated food frequency questionnaire	13.3 years of follow-up; 1,392 endometrial cancer cases	Age, energy intake, ethnicity, education, leisure time physical activity, diabetes status, postmenopausal hormone replacement therapy use, oral contraceptive use, age at first birth, participant in Observational Study, participant in HT trial, participant in DM trial
Xie et al. (11)	US	Cohort study	82,948 female registered nurses	30 to 55 years	Healthy Eating Index -2005, Alternative Healthy Eating Index -2010, and alternate Mediterranean Diet Score	Self-administered semi-quantitative, 131-item, validated food frequency questionnaire	Over 24 years of follow-up; 696 ovarian cancer cases; Ovarian cancer cases were identified either by self-report of the disease on a biennial questionnaire or through family members, the postal service, or the National Death Index	Age, total energy intake, family history of ovarian cancer, tubal ligation, body mass index, parity, number of additional pregnancies, oral contraceptive use duration, smoking (pack-years), menopausal status, type and duration of postmenopausal hormone use, age at menarche, hysterectomy, unilateral oophorectomy, lactose intake, caffeine intake, and physical activity
Chandran et al. (16)	US	Population-based case-control study	205 ovarian cancer cases and 390 controls; Cases were newly diagnosed, pathologically confirmed of invasive epithelial ovarian cancer from the Cancer Registry; Controls were recruited by random digit dialing and confirmed not with a history of hysterectomy and/or bilateral oophorectomy	Mean ages for cases and controls were 57 and 64.6 years	Healthy Eating Index -2005	Block 98.2 food frequency questionnaire for 110 food items	NA	Age, education, race, age at menarche, menopausal status, parity, oral contraceptive use, hormone replacement therapy use, tubal ligation, body mass index, total energy intake, physical activity, smoking status, and pack years smoked
Chandran et al. (17)	US	Population-based case- control study	424 endometrial cancer cases and 398 controls	The mean age was 61.6 years for cases and 64.3 years for controls	Healthy Eating Index -2005	Block 98.2 food frequency questionnaire for 110 food items	NA	Age, education, race, age at menarche, menopausal status and age at menopause for postmenopausal women, parity, oral contraceptive use, hormone replacement therapy use, body mass index, and total calories, physical activity, smoking status, and alcohol
Mai et al. (18)	US	Cohort study	42 254 women	mean age of 61 years	Recommended Foods Score (including information on fruits, vegetables, whole grains, lean meats or meat alternatives, low-fat dairy)	62-item Block/NCI food frequency questionnaire	Median follow-up period of 9.5 years; 142 ovarian cancer cases and 263 endometrial cancer cases; Self-reports of any newly diagnosed cancers. Medical records were obtained to confirm information. Deaths were identified by linkage with the National Death Index and/or the mailed questionnaire.	Age, energy intake, smoking, non-steroidal anti-inflammatory drug use, and body mass index
Harnack et al. (19)	US	Cohort study	34708 postmenopausal women	55 to 69 years	the fifth edition of the Dietary Guidelines for Americans (including fruits, vegetables, grains, milk, meat, saturated fat, cholesterol, total fat, sweetened beverages, sodium, alcohol weight, physical activity)	Self-administered, validated food frequency questionnaire for 127 food items	13 year of follow-up; 164 ovarian cancer cases; Cancer diagnosis was ascertained through the State Health Registry of Iowa	Age, energy intake, smoking status, pack-years of cigarette smoking, age at menopause, family history of ovarian cancer in first-degree relatives, nulliparity

^1^Healthy Eating Index-2005 includes components of total fruit (included 100% juice), whole fruit (not juice), total vegetables, dark green and orange vegetables and legumes, total grains, whole grains, milk, meat and beans, oils, saturated fat, sodium, calories from solid fat, alcohol and added sugar; Healthy Eating Index-2010 includes components of total fruit (all forms of fruit, including fruit juice), whole fruit (all forms except fruit juice), total vegetables, greens and beans, whole grains, dairy, total protein foods (lean portion of meat and poultry; eggs; beans and peas), seafood and plant proteins, fatty acids (ratio of polyunsaturated and monounsaturated fatty acids to saturated fatty acids), refined grains, sodium, empty calories (calories from solid fats, alcohol and added sugars); Alternative Healthy Eating Index-2010 includes components of fruit, vegetables, whole grains, soda and fruit juice, nuts and legumes, processed/red meat, trans fat, long-chain (n-3) fats, polyunsaturated fatty acids, sodium and alcohol; Mediterranean Diet Score includes components of vegetables (excluding potatoes), fruit (including juices), legumes, nuts, fish, ratio of monounsaturated fat to saturated fat, red and processed meats and alcohol; Dietary Approaches to Stop Hypertension includes components of fruit (all fruits and fruit juice), vegetables (all vegetables except potatoes and legumes), nuts and legumes, whole grains, low-fat dairy, sodium, red and processed meats, sweetened beverages.

### Diet Quality and Ovarian Cancer Risk

Seven studies evaluated the association of diet quality with risk of ovarian cancer. Two studies reported significantly inverse association of diet quality as assessed by AHEI-2010 and HDS with risk of ovarian cancer (Qin et al., 2017 study (10)): RR _AHEI-2010_ = 0.66, 95%CI: 0.45, 0.98; Arthur et al. (12) study: RR _HDS_ = 0.50, 95%CI: 0.27, 0.92). One study reported significantly increased risk of ovarian cancer associated with high quality diet as assessed by “Dietary Guidelines for Americans Index” (RR = 2.05, 95%CI: 1.19, 3.52). Four studies reported null associations with RRs ranged from 0.76 (95%CI: 0.47, 1.22) to 1.03 (95%CI: 0.80, 1.34) for other diet quality indices such as HEI-2005, HEI-2010, aMDS and RFS. The main results for these seven included studies were shown in **Table 2**.

**Table 2:** Main results for ovarian cancer in the original studies*.

Study	Index	Relative Risk (95%CI)
Arthur et al. (9)	Diet Score Quintiles	
	≤20	Reference
	21–25	1.15 (0.93, 1.43)
	26–29	1.21 (0.96, 1.52)
	30–34	1.16 (0.92, 1.47)
	>34	1.26 (0.99, 1.62)
	Healthy Diet Score	
	< 24	Reference
	24-27	0.48 (0.25, 0.92)
	27-31	0.76 (0.44, 1.29)
	31-35	0.66 (0.36, 1.22)
	**>35**	**0.50 (0.27, 0.92)**
Qin et al. (10)	Healthy Eating Index-2005	
	47.8	Reference
	57.6	0.87 (0.60, 1.26)
	64.8	1.03 (0.70, 1.50)
	73.5	0.83 (0.56, 1.23)
	Healthy Eating Index-2010	
	48.4	Reference
	58.4	0.86 (0.60, 1.25)
	65.5	0.81 (0.55, 1.19)
	75.4	0.74 (0.50, 1.11)
	Alternate Healthy Eating Index-2010	
	41.4	Reference
	49.1	0.83 (0.57, 1.19)
	55.2	0.83 (0.57, 1.19)
	**67.9**	**0.66 (0.45, 0.98)**
Xie et al. (11)	Alternate Healthy Eating Index-2010	
	≤42	Reference
	>42-48	1.02 (0.78, 1.32)
	>48-53	1.23 (0.96, 1.59)
	>53-59	1.11 (0.86, 1.43)
	>59	1.03 (0.80, 1.34)
	Healthy Eating Index-2005	
	≤56	Reference
	>56-63	0.79 (0.61, 1.03)
	>63-68	0.82 (0.63, 1.06)
	>68-74	0.93 (0.72, 1.20)
	>74	0.85 (0.65, 1.12)
	Alternate Mediterranean Diet Score	
	≤2.6	Reference
	>2.6-3.5	0.90 (0.70, 1.16)
	>3.5-4.5	0.83 (0.65, 1.05)
	>4.5-5.5	0.85 (0.66, 1.09)
	>5.5	0.91 (0.71, 1.18)
Chandran et al. (16)	Healthy Eating Index-2005	
	Tertile 1 (<67.39)	Reference
	Tertile 2 (67.39–74.50)	0.86 (0.54, 1.40)
	Tertile 3 (≥74.51)	0.90 (0.55, 1.47)
Mai et al. (18)	Recommended Foods Score	
	6.4	Reference
	10	0.84 (0.54, 1.33)
	12.5	0.80 (0.48, 1.30)
	16	0.76 (0.47, 1.22)
Harnack et al. (19)	Dietary Guideline Index	
	7.1 (2.1-8.3)	Reference
	9.0 (8.4-9.6)	1.59 (0.91, 2.78)
	10.2 (9.7-10.8)	1.94 (1.13, 3.32)
	11.5 (10.9-12.1)	1.79 (1.03, 3.09)
	13.5 (12.2-17.6)	2.05 (1.19, 3.52)

*The bold values were statistically significant in the original studies.

In the dose-response meta-analysis of three studies assessing diet quality with HEI-2005 index, the pooled RR for each 10 points increase was 0.98 (95%CI: 0.95, 1.01) without statistical heterogeneity (I^2^ = 0%, P _heterogeneity_ = 0.968). Two studies assessed the diet quality using AHEI-2010 index, with pooled RR of 0.94 (95%CI: 0.77, 1.13) for each 10 points increase. Since limited number of studies were included in the dose-response results, these non-significant results should still be interpreted with caution due to lack of power.

### Diet Quality and Endometrial Cancer Risk

Seven studies evaluated the association of diet quality with risk of endometrial cancer. Three studies reported significantly inverse association of diet quality as assessed by the MDS and Diet Score Quintiles with risk of endometrial cancer (Ricceri et al. (13) study: RR _MDS_ = 0.51, 95%CI: 0.28, 0.92; Filomeno et al. (14) study: RR _MDS_ = 0.43, 95%CI: 0.34, 0.56; Arthur et al. (9) study: RR _Diet Score Quintiles_ = 0.81, 95%CI: 0.67, 0.98). Four studies reported null association with RRs ranged from 0.83 (95%CI: 0.52, 1.34) to 0.98 (95%CI: 0.82, 1.17) for other diet quality indices including HEI-2005, HEI-2010, RFS and HDS. The main results for the seven included studies were shown in **Table 3**. In the dose-response meta-analysis of two studies assessing diet quality with the MDS, the pooled RR for one unit increment of the MDS was 0.87 (95%CI: 0.81, 0.93).

**Table 3:** Main results for endometrial cancer in the original studies*.

Study	Index	Relative Risk (95%CI)
Arthur et al. (12)	Diet Score Quintiles	
	≤20	Reference
	21–25	0.97 (0.83, 1.13)
	**26–29**	**0.79 (0.66, 0.94)**
	30–34	0.85 (0.71, 1.02)
	**>34**	**0.81 (0.67, 0.98)**
	Healthy Diet Score	
	< 24	Reference
	24-27	1.61 (1.00, 2.60)
	27-31	1.17 (0.72, 1.91)
	31-35	1.50 (0.91, 2.49)
	>35	0.91 (0.53, 1.56)
Ricceri et al. (13)	Mediterranean Diet Score	
	0–3	Reference
	**4–5**	**0.57 (0.39, 0.86)**
	**6–8**	**0.51 (0.28, 0.92)**
Filomeno et al. (14)	Mediterranean Diet Score	
	0 to 3	Reference
	4	0.82 (0.67, 1.00)
	**5**	**0.66 (0.54, 0.81)**
	**6**	**0.54 (0.43, 0.68)**
	**7 to 9**	**0.43 (0.34, 0.56)**
George et al. (15)	Healthy Eating Index-2010	
	Q1	Reference
	Q2	1.17 (0.99, 1.37)
	Q3	1.01 (0.84, 1.20)
	Q4	1.02 (0.85, 1.22)
	Q5	1.11 (0.93, 1.33)
	Alternate Healthy Eating Index-2010	
	Q1	Reference
	Q2	0.96 (0.82, 1.14)
	Q3	1.09 (0.93, 1.29)
	Q4	0.97 (0.82, 1.15)
	Q5	0.98 (0.82, 1.17)
	Alternate Mediterranean Diet Score	
	Q1	Reference
	Q2	0.96 (0.79, 1.15)
	Q3	1.05 (0.88, 1.26)
	Q4	1.04 (0.87, 1.25)
	Q5	0.98 (0.82, 1.17)
	Dietary Approaches to Stop Hypertension	
	Q1	Reference
	Q2	0.99 (0.83, 1.20)
	Q3	1.10 (0.94, 1.28)
	Q4	1.07 (0.89, 1.28)
	Q5	1.00 (0.84, 1.19)
Chandran et al. (17)	Healthy Eating Index-2005	
	<66.30	Reference
	66.30–72.48	1.21 (0.70, 1.88)
	72.49–77.98	1.32 (0.85, 2.06)
	≥77.99	0.83 (0.52, 1.34)
Mai et al. (18)	Recommended Foods Score	
	6.4	Reference
	10	0.78 (0.55, 1.10)
	12.5	0.91 (0.63, 1.30)
	16	0.87 (0.61, 1.22)

*The bold values were statistically significant in the original studies.

## Discussion

This review of epidemiological studies suggested little clear association of major diet quality indices, including HEI-2005, HEI-2010, AHEI-2010, aMDS and RFS, with risk of ovarian cancer. Of note, adherence to higher quality of diets, as assessed by MDS, may be associated with a lower risk of endometrial cancer. Dose-response analysis suggested a 13% reduction in risk of endometrial cancer for one unit increment in MDS in women. However, this inverse association with endometrial cancer was not observed for other diet quality indices including HEI-2005, HEI-2010 and RFS etc.

Assessment of the overall quality of diet instead of single nutrients can address the likely interaction between multiple diet components (5). Previous meta-analyses suggested high quality diets as assessed by the HEI, AHEI or DASH were associated with a significant reduction in the risk of total cancer (6, 36). However, concerning the ovarian cancer, the results were inconclusive since there is only one epidemiological study included in the previous study, and reported a null association (6). In the current review of seven studies, most studies still reported a null association. Although Qin et al., 2007 reported a reduced risk of ovarian cancer associated with the highest category of AHEI-2010 index, such significantly inverse association was not observed in the further dose-response meta-analysis. In addition, a population-based cohort study in China where the diet habits and pattern were different from the Western countries suggested that diet quality as assessed by Chinese food pagoda and modified DASH score was not associated with cancers in breast, uterus, cervix, placenta, or ovary combined, while adherence to modified AHEI-2010 was significantly associated with lower risk of these cancers combined (35). Compared with ovarian cancer, breast cancer cases accounted for a larger proportion. Thus, the inverse association was more likely to be due to a reduced risk of breast cancer rather than the ovarian cancer (35). Taken together, there was still no clear evidence indicating any association of diet quality with risk of ovarian cancer.

Our results suggested that, compared with other dietary indices HEI-2005, HEI-2010 and AHEI-2010, the MDS may capture aspects of diet more relevant to endometrial cancer. In a large case-control study in Italian population, there was a reduced risk of endometrial cancer for increasing adherence to the Mediterranean diet (14). The Mediterranean diet contains several antioxidants with important anti-inflammatory properties that have been inversely related with cancer risk (37). The Mediterranean diet is rich in vitamins, carotenoids, flavonoids and folates (mainly derived from vegetables and fruits), which have shown inverse relations with endometrial cancer in case-control studies (13, 14). Moreover, a large proportion of energy intake in the Mediterranean diet derives from cereals and other plant sources rather than from animal sources, which has been inversely associated with endometrial cancer risk (38). Besides, the richness of phytoestrogens in soy and whole grain products from the Mediterranean diet may exert protective effect on endometrial cancer (39). Since MDS takes into account the interactions among various combinations of foods and nutrients and their synergistic effects, Mediterranean diet as a whole may be a stronger determinant of endometrial cancer risk than the single dietary components (14).

There are several limitations in the study. Despite there are certain commonalities on the diet components for these diet quality indices, the weights of each components, assessment methods, and categories of the diet indices varied from study to study. The diet quality indices in these studies included HEI-2005, HEI-2010, AHEI-2010, aMDS and RFS, etc., Due to high heterogeneity of the diet quality as assessed by various diet quality indices, this review was mainly based on a systematic review of the current evidence, and little quantitative conclusion could be drawn. Although the dose-response meta-analyses for certain dietary indices were performed, the pooled risk estimates, especially the non-significant results, were still inconclusive due to the inclusion of a limited number of epidemiological studies. These results should still be interpreted with caution due to lack of statistical power. Further evidence from well-designed, large-scale cohort studies are still warranted before we could draw a confirmative conclusion. Furthermore, the epidemiological studies in the review were conducted in the Western population. As such, we should be more cautious in generalizing the results to other areas, especially where the dietary patterns and habits are different, such as Asia and Africa. In addition, non-English databases were not searched in the review, which may lead to publication bias.

In conclusion, this systematic review of the epidemiological studies suggests little evidence on the association between diet quality and risk of ovarian cancer. Adherence to high quality diets, as assessed by MDS, may be associated with lower risk of endometrial cancer. If a causal association could be confirmed by further well-designed studies, the adherence of the Mediterranean diet guidance might be encouraged for the prevention of endometrial cancer in women.

## Data Availability Statement 

The datasets during and/or analyzed during the current study are available from the corresponding author on reasonable request. Requests to access these datasets should be directed to fusan2020@sina.com.

## Author Contributions 

Y-HZ developed the research design, interpreted the results, and also had primary responsibility for the final content. ZL and M-ZT analyzed the data and interpreted the results. Y-HZ drafted manuscript. All authors contributed to the article and approved the submitted version.

## Conflict of Interest

The authors declare that the research was conducted in the absence of any commercial or financial relationships that could be construed as a potential conflict of interest.
