# Supplementary material for: Association Between Diet Quality and Risk of Ovarian and Endometrial Cancers: A Systematic Review of Epidemiological Studies
Source: Front Oncol. 2021 May 18;11:659183. doi: 10.3389/fonc.2021.659183 (PMC8168438; doi:10.3389/fonc.2021.659183)
Supplement: Supplementary file 2 [file Table_2.docx]

**Literature search strategy for the systematic review**

1. **PubMed (n = 66)**

("diet quality"[All Fields] OR "diet score"[All Fields] OR "diet index"[All Fields] OR "healthy eating index"[All Fields] OR "Mediterranean diet"[All Fields] OR "dietary guidelines"[All Fields] OR "lifestyle index"[All Fields]) AND ("ovarian"[All Fields] OR "ovarians"[All Fields] OR ("ovarial"[All Fields] OR "ovary"[MeSH Terms] OR "ovary"[All Fields] OR "ovaries"[All Fields] OR "ovary s"[All Fields]) OR "endometrial"[All Fields] OR ("endometrium"[MeSH Terms] OR "endometrium"[All Fields] OR "endometriums"[All Fields])) AND ("cancer s"[All Fields] OR "cancerated"[All Fields] OR "canceration"[All Fields] OR "cancerization"[All Fields] OR "cancerized"[All Fields] OR "cancerous"[All Fields] OR "neoplasms"[MeSH Terms] OR "neoplasms"[All Fields] OR "cancer"[All Fields] OR "cancers"[All Fields] OR ("cysts"[MeSH Terms] OR "cysts"[All Fields] OR "cyst"[All Fields] OR "neurofibroma"[MeSH Terms] OR "neurofibroma"[All Fields] OR "neurofibromas"[All Fields] OR "tumor s"[All Fields] OR "tumoral"[All Fields] OR "tumorous"[All Fields] OR "tumour"[All Fields] OR "neoplasms"[MeSH Terms] OR "neoplasms"[All Fields] OR "tumor"[All Fields] OR "tumour s"[All Fields] OR "tumoural"[All Fields] OR "tumourous"[All Fields] OR "tumours"[All Fields] OR "tumors"[All Fields]) OR ("neoplasm s"[All Fields] OR "neoplasms"[MeSH Terms] OR "neoplasms"[All Fields] OR "neoplasm"[All Fields]) OR ("carcinoma"[MeSH Terms] OR "carcinoma"[All Fields] OR "carcinomas"[All Fields] OR "carcinoma s"[All Fields]))

1. **Embase (n = 112)**

('diet quality'/exp OR 'diet quality' OR 'diet score' OR 'diet index' OR 'healthy eating index'/exp OR 'healthy eating index' OR 'mediterranean diet'/exp OR 'mediterranean diet' OR 'dietary guidelines' OR 'lifestyle index') AND (ovarian OR 'ovary'/exp OR ovary OR endometrial OR 'endometrium'/exp OR endometrium) AND ('cancer'/exp OR cancer OR 'tumor'/exp OR tumor OR 'neoplasm'/exp OR neoplasm OR 'carcinoma'/exp OR carcinoma)

1. **Web of Science (n = 86)**

TOPIC: (“Diet quality” OR “Diet Score” OR “Diet Index” OR “Healthy Eating Index” OR “Mediterranean Diet” OR “Dietary Guidelines” OR “lifestyle index”) AND TOPIC: (Ovarian OR Ovary OR Endometrial OR Endometrium) AND TOPIC: (Cancer OR Tumor OR Neoplasm OR Carcinoma)

Timespan: All years. Indexes: SCI-EXPANDED, CPCI-S, CCR-EXPANDED, IC.

1. **Scopus (n = 15)**

(TITLE-ABS-KEY ( “diet AND quality” OR “diet AND score” OR “diet AND index” OR “healthy AND eating AND index” OR “mediterranean AND diet” OR “dietary AND guidelines” OR “lifestyle AND index” ) AND TITLE-ABS-KEY ( ovarian OR ovary OR endometrial OR endometrium ) AND TITLE-ABS-KEY ( cancer OR tumor OR neoplasm OR carcinoma ) )
