# Supplementary material for: Association Between Diet Quality and Risk of Ovarian and Endometrial Cancers: A Systematic Review of Epidemiological Studies
Source: Front Oncol. 2021 May 18;11:659183. doi: 10.3389/fonc.2021.659183 (PMC8168438; doi:10.3389/fonc.2021.659183)
Supplement: Supplementary file 3 [file Table_3.docx]

**Table S2 Study quality of cohort studies included in the review ^1^**

| **Study** | **Selection** | | | | **Comparability** | **Outcome** | | | **Total score** |
| --- | --- | --- | --- | --- | --- | --- | --- | --- | --- |
|  | **Representativeness of the exposed cohort** | **Selection of the unexposed cohort** | **Ascertainment of exposure** | **Outcome of interest not present at start of study** | **Control for potential confounders^2^** | **Assessment of outcome** | **Follow-up long enough for outcomes to occur^3^** | **Adequacy of follow-up of cohorts ^4^** |  |
| Arthur, 2019 | ⚝ | ⚝ | ⚝ | ⚝ | ⚝⚝ | ⚝ | ⚝ | ⚝ | 9 |
| Arthur, 2018 | ⚝ | ⚝ | ⚝ | ⚝ | ⚝⚝ | ⚝ | ⚝ | ⚝ | 9 |
| George, 2015 | ⚝ | ⚝ | ⚝ | ⚝ | ⚝⚝ | ⚝ | ⚝ | ⚝ | 9 |
| Xie, 2014 | - | ⚝ | ⚝ | ⚝ | ⚝⚝ | ⚝ | ⚝ | ⚝ | 8 |
| Mai, 2005 | ⚝ | ⚝ | ⚝ | ⚝ | ⚝⚝ | ⚝ | ⚝ | ⚝ | 9 |
| Harnack, 2002 | ⚝ | ⚝ | ⚝ | ⚝ | ⚝⚝ | ⚝ | ⚝ | ⚝ | 9 |

^1^ In this review, a study with a NOS score of ≥7 was considered as high-quality study with low risk of bias. Thus, all the cohort studies in the review were high-quality studies.

^2^ A maximum of 2 stars could be awarded for this item. Studies that controlled for age and total energy intake received one star, whereas studies that controlled for most of the other important confounders such as body mass index, tobacco smoke, family history, physical activity or menopausal status, etc. received an additional star.

^3^ A cohort study with a follow-up time >3 years was assigned one star.

^4^ A cohort study with subjects lost to follow up unlikely to introduce bias, or a follow-up rate >75% was assigned one star.

**Table S3 Study quality of case-control studies included in the review ^1^**

| **Study** | **Selection** | | | | **Comparability** | **Exposure** | | | **Total Score** |
| --- | --- | --- | --- | --- | --- | --- | --- | --- | --- |
|  | **Is the case definition adequate** | **Representativeness of the cases** | **Selection of controls** | **Definition of controls** | **Control for potential confounders^2^** | **Ascertainment of exposure** | **Same method of ascertainment for cases and controls** | **Non-Response rate** |  |
| Qin, 2017 | ⚝ | ⚝ | ⚝ | ⚝ | ⚝⚝ | ⚝ | ⚝ | - | 8 |
| Ricceri, 2017 | ⚝ | ⚝ | - | ⚝ | ⚝⚝ | ⚝ | ⚝ | ⚝ | 8 |
| Filomeno, 2015 | ⚝ | ⚝ | - | ⚝ | ⚝⚝ | ⚝ | - | ⚝ | 7 |
| Chandran, 2011 | ⚝ | - | ⚝ | ⚝ | ⚝⚝ | ⚝ | ⚝ | - | 7 |
| Chandran, 2010 | ⚝ | - | ⚝ | ⚝ | ⚝⚝ | ⚝ | ⚝ | - | 7 |

^1^ In this review, a study with a NOS score of ≥7 was considered as high-quality study with low risk of bias. Thus, all the case-control studies in the review were high-quality studies.

^2^ A maximum of 2 stars could be awarded for this item. Studies that controlled for age and total energy intake received one star, whereas studies that controlled for most of the other important confounders such as body mass index, tobacco smoke, family history, physical activity or menopausal status, etc. received an additional star.
